# Cerebral venous sinus thrombosis in a patient with *Klebsiella pneumoniae* primary liver abscess: a case report

**DOI:** 10.1186/s12883-022-02806-y

**Published:** 2022-07-30

**Authors:** Lingyu Zhou, Chao Wang, Jialan Bian, Siyuan Xu, Minjie Yang, Mingquan Chen

**Affiliations:** 1 Emergency Department and Department of Infectious Diseases, Huashan Hospital of Fudan University, Shanghai, 200040 China; 2 Department of Infectious Diseases, Huashan Hospital of Fudan University, Shanghai, China

**Keywords:** Cerebral venous sinus thrombosis, Liver abscess, Case report

## Abstract

**Background:**

Liver abscess is a common emergency in the emergency department. However, cerebral venous sinus thrombosis (CVST) is a rare and serious cerebrovascular disease. Cases of CVST in patients with *Klebsiella pneumoniae* primary liver abscess (KLA) have not been described in the literature. We report a case of CVST in patients with KLA.

**Case presentation:**

A 54-year-old male patient came to our department with a fever for 2 days and altered mental status for 1 day. Abdominal computed tomography (CT) and liver magnetic resonance imaging (MRI) revealed multiple liver abscesses. The blood culture was identified as *Klebsiella pneumoniae* sepsis. Head contrast-enhanced MRI and magnetic resonance venography (MRV) imaging showed multiple thrombus formation in the right transverse sinus and sigmoid sinus. The patient's infection and thrombosis were controlled within one week of multidisciplinary comprehensive treatment such as antibiotic and antithrombotic therapy, and a good clinical recovery during the 1-month follow-up.

**Conclusion:**

CVST after liver abscess is rare, clinicians should be aware of this complication and vigilant for the possibility of bacterial meningitis. The underlying mechanisms need to be further studied.

## Background

Acute liver abscess is the most common type of visceral abscess, accounting for about 48% of visceral abscess [1]. The annual incidence rate is estimated at 2.3 cases per 100,000 people and is higher among men than that in women [2]. About 80% of liver abscesses are caused by bacteria, mainly enteric gram-negative bacilli and anaerobic bacteria. In Mainland China, more than 80% of liver abscess cases are caused by *Klebsiella pneumoniae* [3]. In addition to typical bacterial liver abscess manifestations (such as fever, chills, right upper abdominal pain, elevated white blood cells, and liver enzymes), some cases may also present "invasion syndrome", including endophthalmitis, central nervous system infection, other extra-hepatic organ infection, and multiple organ dysfunction (MODS), etc. [4]. Compared with other bacteria, KLA is more likely to cause septic thrombophlebitis of portal vein or hepatic vein system [5]. Inferior vena cava thrombosis in patient with KLA has also been rarely reported [6].

Cerebral venous sinus thrombosis (CVST) is a rare but serious cerebrovascular disease with an annual incidence of 0.22 to 1.57 per 100,000 [7, 8]. Different from arterial stroke, CVST is more likely to occur in young adults and children, especially females [9]. International studies on Cerebral Vein and Dural Sinus Thrombosis (ISCVT) showed that the median age of patients with CVST was 37 years [10]. CVST can be caused by a variety of medical conditions, and the major risk factors in adults include genetic or acquired thrombogenic conditions, oral contraceptives, pregnancy and puerperium, malignant tumor, infection, inflammatory diseases, head injury and mechanical causes [9].

A small number of patients with KLA have concurrent evidence of or develops metastatic infection at other sites [2, 11, 12]. Although infectious disease is one of the risk factors for CVST, when we encounter KLA patients with persistent fever or signs and symptoms of superior brain function disturbances, clinicians are more likely to think about the occurrence of metastatic infection, such as endophthalmitis, meningitis and brain abscess. CVST is a rare complication that can be easily overlooked. In this article, we report a case of transverse sinus and sigmoid sinus thrombosis with KLA. The patient’s consent was obtained for publishing this case report, and the corresponding written informed consent was signed.

## Case presentation

A 54-year-old male patient came to our department due to "fever for 2 days accompanied by altered mental status for 1 day". The patient did not have obvious gastrointestinal symptoms neither did he complain of headache or vision difficulties. He developed fever and chills without obvious inducement 2 days ago. The maximum body temperature was 40 Degrees Celsius, body temperature is often above 39 Degrees Celsius, with a large fluctuation range. One day before admission, the patient had sudden altered mental status for one time, which was manifested as difficulty in answering questions, and resolved spontaneously after half an hour. The patient was admitted to a local hospital and performed some laboratory tests (Table 1). The next day, he was transferred to our hospital by ambulance. The patient denied any history of trauma or other significant medical history. And the patient's epidemiological history for COVID-19 was negative, as well as the COVID tests. The patient had not received any COVID-19 vaccine at the time.

**Table 1:** Patient's laboratory results during the in-hospital stay

Patient's Laboratory finding (normal range)	The day before admission	On admission	The third day of admission	The sixth day of admission	Follow-up one month
WBC, (3.5–9.5 × 10^9^/L)	4.58	10.21	10.40	8.09	5.96
PLA, (125–350 × 10^9^/L)	53	35	26	112	221
Neutrophil%, (40.0–75.0%)	90.7	86.3	80.1	76.8	62.8
CRP, (< 8.20 mg/L)	320.42	245.22	188.71	92.23	0.98
PCT, (≤ 0.05 ng/mL)	-	> 100	> 100	16.7	0.06
LDH, (125-247U/L)	-	569	362	232	171
AMY, (30-110U/L)	-	94	144	60	180
GLU, (3.9–5.8 mmol/L)	-	7.3	6.7	7.7	5.9
ALT, (9-50U/L)	92	206	105	53	27
AST, (15-40U/L)	165	366	92	25	25
TBIL, (≤ 26.0 μmol/L)	38.7	33.6	27.4	14.6	6.5
SCr, (57-97 μmol/L)	142	120	71	53	57
BUN, (3.1–8.0 mmol/L)	-	9.7	6.1	3.3	4.3
ALB, (40-55 g/L)	-	40	30	31	47
INR, (0.92–1.15)	1.33	1.04	1.17	-	1.02
DDI, (≤ 0.55 mg/L)	42.9	4.98	4.09	-	< 0.19
TnT, (0.013–0.025 ng/ml)	0.025	0.043	0.027	-	0.006
NT-pro BNP, (< 60.4 pg/mL)	-	900.2	1481.0	-	108.2
LAC, (0.63–2.50 mmol/L)	-	2.0	0.74	-	1.2
**CSF test**					
Pressure, (80–180 mmH_2_O)	-	-	120mmH_2_O	-	-
CSF protein, (120–600 mg/L)	-	-	350	-	-
CSF WBC count, (0–8 × 10^6^/L)	-	-	2	-	-
CSF glucose, (2.5–4.5 mmol/L)	-	-	3.8	-	-
CSF Cl^−^ (120–132 mmol/L)	-	-	125	-	-

*WBC* White blood cell, *PLA* Platelets, *CRP* C-reactive protein, *PCT* Procalcitonin, *LDH* Lactate dehydrogenase, *AMY* Amylase, *GLU* serum glucose, *ALT* Alanine aminotransferase, *AST* Aspartate transaminase, *TBIL* Total bilirubin, *SCr* Serum creatinine, *BUN* Blood urea nitrogen, *ALB* serum albumin, *INR* International normalized ratio, *DDI* D dimer, *TnT* Troponin T, *NT-pro BNP* N-terminal pro-brain natriuretic peptide, *LAC* Lactic acid, *CSF* Cerebrospinal fluid

**Fig. 1:**
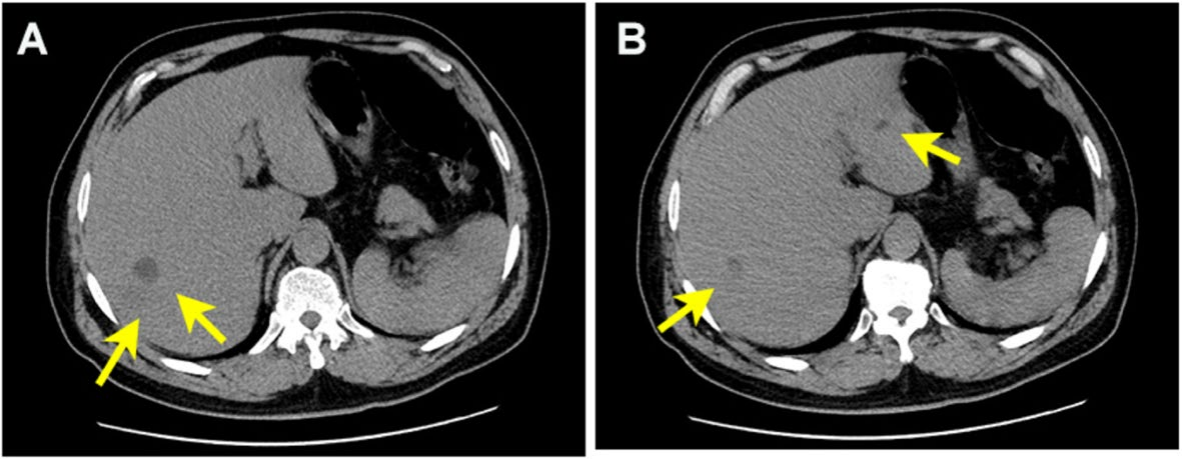
**A** and **B** Abdominal computed tomography scan revealed a patchy, slightly low-density area in the lower right posterior lobe of the liver (*arrows*), with an area of about 55 × 45 mm

On admission, no positive signs were found on neurological examination. Physical examination revealed positive percussion pain in the liver area. Abdominal ultrasound showed a 46 × 37 mm slightly hypoechoic area on the right posterior lobe of the liver. Portal vein ultrasound did not reveal significant thrombus formation. Abdominal CT examination also revealed a patchy, slightly low-density area in the lower right posterior lobe of the liver (Fig. 1). Head CT without contrast did not find any abnormalities. Laboratory tests showed in Table 1.

**Fig. 2:**
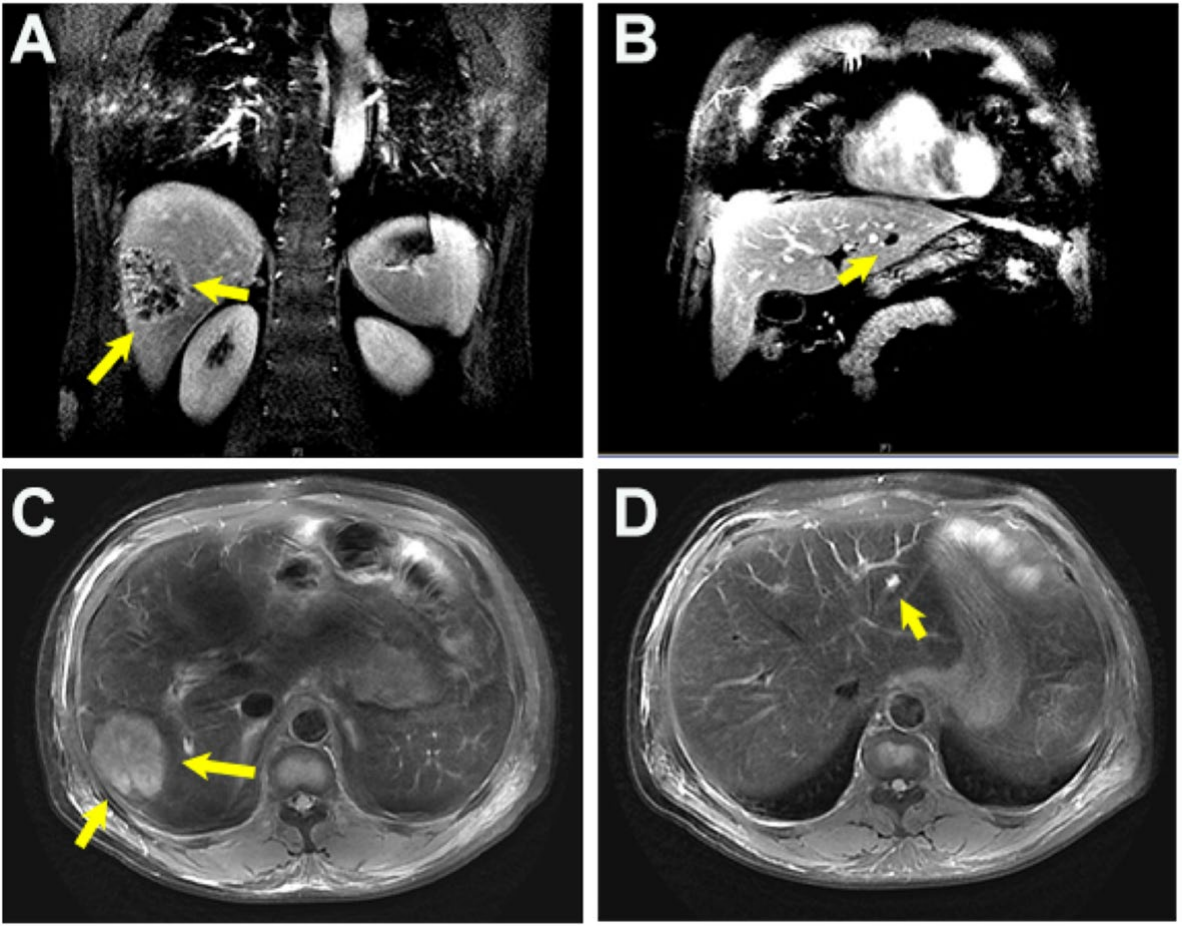
**A**-**D** The enhanced magnetic resonance imaging of the liver showed multiple round-like abnormal signals in the liver. After the enhancement, there was obvious uneven ring enhancement. The larger one (*arrow*) was located in the right lobe of the liver and was about 59 × 49 mm in size, suggesting multiple abscesses in the liver

After considering the initial diagnosis of sepsis and liver abscess, we immediately administered empiric anti-infective therapy, as well as the necessary stable internal environment and organ support treatment after blood culture. Further enhancement of liver MRI suggested the formation of multiple abscesses in the liver (Fig. 2). Then the blood culture was identified as *Klebsiella pneumoniae*. After comprehensive evaluation together with the surgeon, we decided to continue antibacterial treatment and temporarily postpone drainage for two reasons. The first is that the abscesses did not completely liquefy according to the perspective of imaging. Secondly, the largest abscess formed a septum so that adequate drainage cannot be achieved.

**Fig. 3:**
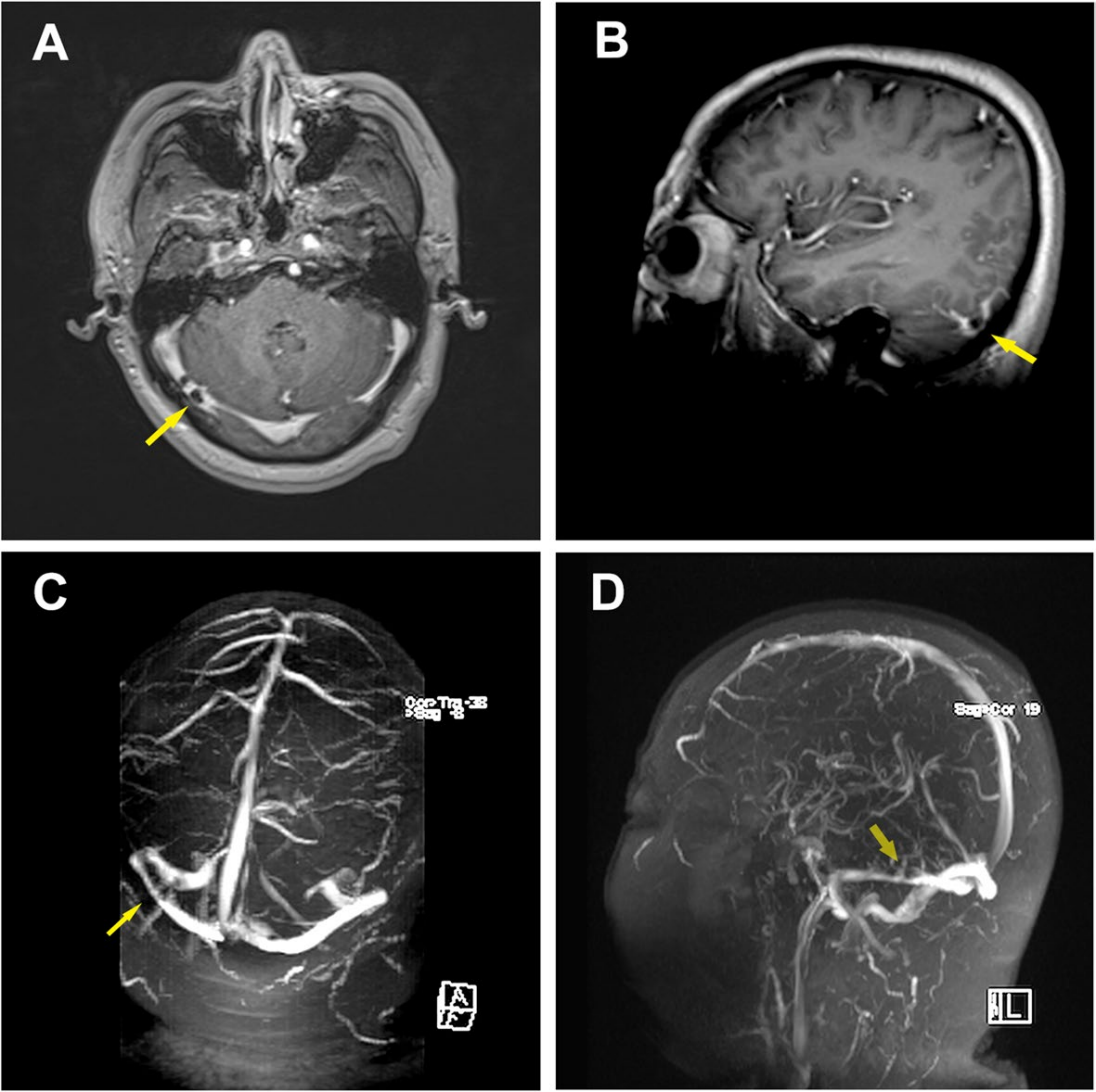
**A** and **B** Contrast-enhanced magnetic resonance imaging revealed the suspicious filling defect of the right transverse sinus, and the larger one (*arrow*) was about 6 mm, suggesting the possibility of thrombosis in the right transverse sinus. **C** and **D** Magnetic resonance venography imaging showed multiple filling defects in the right transverse sinus and sigmoid sinus (*arrow*), and the luminal visualization was slender than that on the left (*arrow*), suggesting multiple thrombosis in the right transverse sinus and sigmoid sinus

Assessing systemic organ involvement and the dissemination of abscesses, no intraocular dissemination was found during ophthalmological examination. Head contrast-enhanced MRI showed thrombosis of the right transverse sinus (Fig. 3A and B). To clarify whether there is thrombosis in the cerebral venous sinus, the head MRV was performed. MRV revealed multiple filling defects in the right transverse sinus and sigmoid sinus, and the luminal visualization was slender, suggesting multiple thrombosis in the right transverse sinus and sigmoid sinus (Fig. 3C and D). Based on these results, CVST could be diagnosed. After that, the lumbar puncture (LP) was perfected to rule out cerebral infections on the third day of admission. The results of cerebrospinal fluid (CSF) were shown in Table 1. Subsequently, CSF cultures of pathogenic microorganisms (bacteria, fungi and mycobacterium tuberculosis) were also negative.

**Fig. 4:**
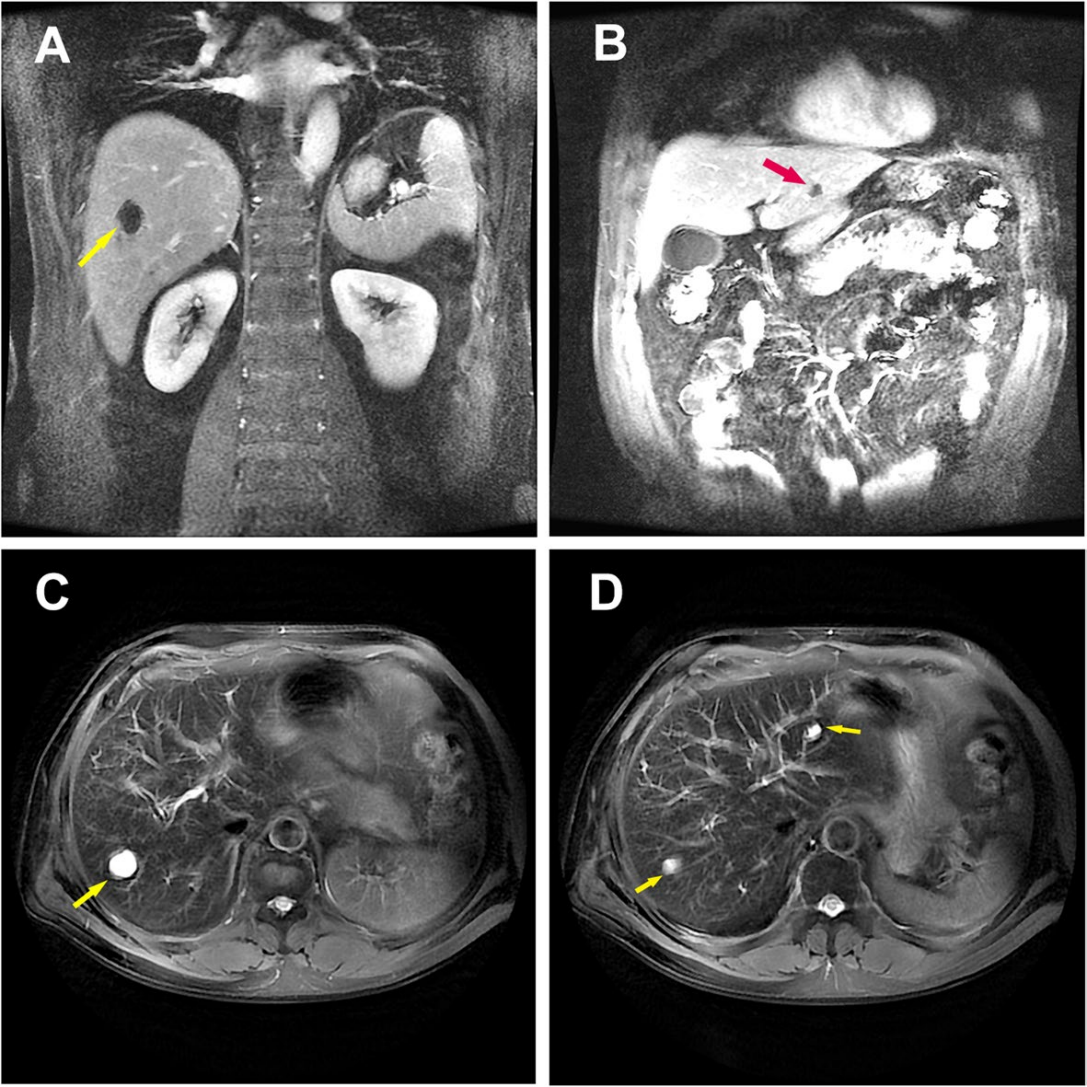
**A**-**D** Enhanced magnetic resonance imaging of the liver obtained at 1-month follow-up showed that lumpy abnormal signal (*arrow*) was observed in the right posterior lobe of the liver, about 28 × 31 mm in size, which was reduced compared with that of the previous one

**Fig. 5:**
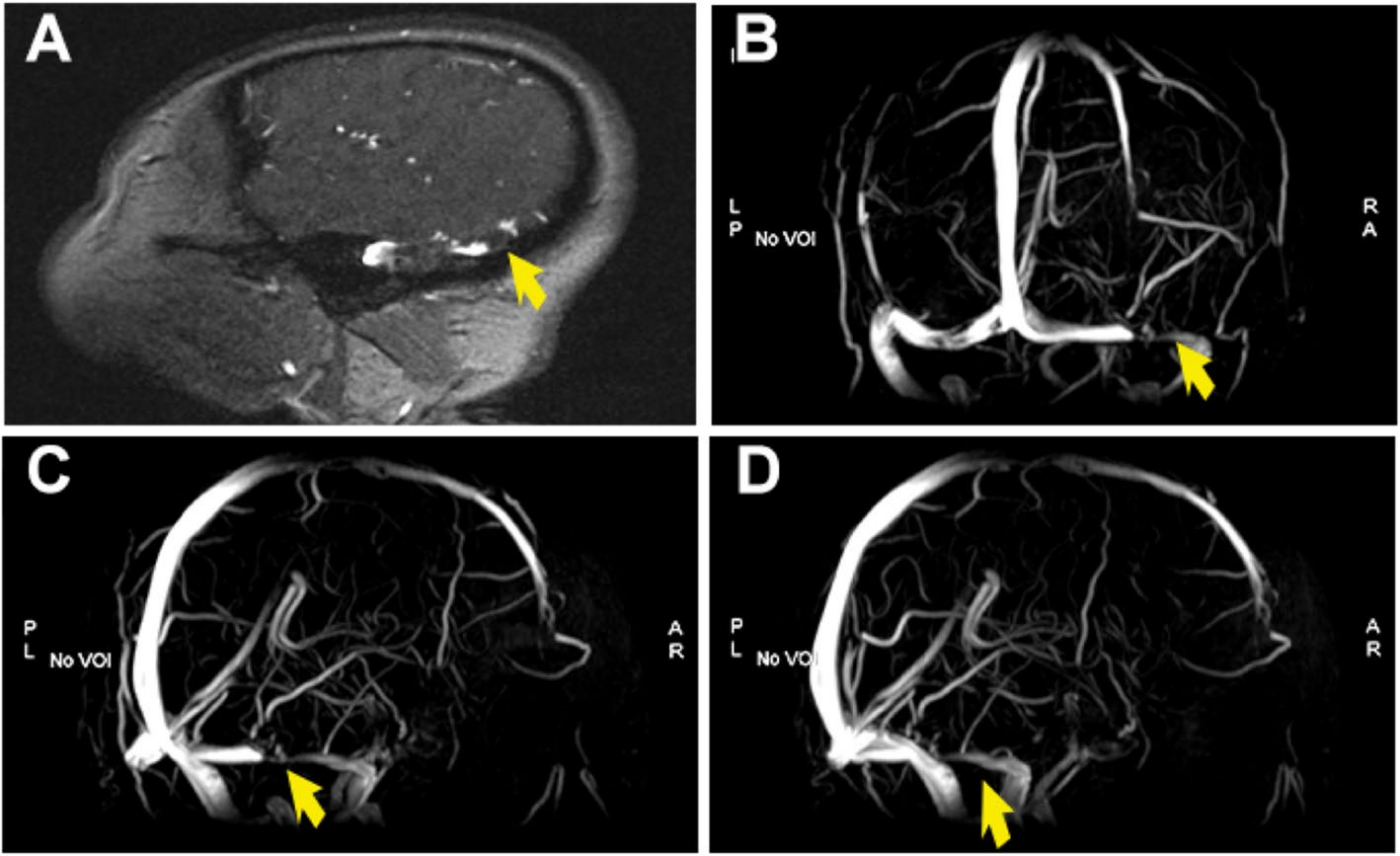
**A**-**D** Magnetic resonance imaging obtained at 1-month follow-up showed no significant change in the right transverse and sigmoid sinus (*arrow*)

In the end, the main diagnosis was “1. Sepsis, 2. *Klebsiella pneumoniae* primary liver abscess, 3. Cerebral venous sinus thrombosis.” According to drug susceptibility and renal function, the antibacterial program was adjusted to combined antibacterial regimen. For CVST, subcutaneous injection of low-molecular-weight heparin (LMWH) was used as anticoagulant therapy (nadroparin calcium 8200 IU/day). After comprehensive treatment, the patient's body temperature dropped to normal without altered mental status or headache. Also, the blood routine returned to normal, liver and kidney function and other biochemical indicators improved significantly (Table 1). The total course of antibiotic treatment was 8 weeks. The specific plan was as follows: (6 weeks of intravenous therapy, the first 2 weeks of intensive therapy was imipenem cilastatin sodium + amikacin + metronidazole, and then the subsequent treatment step was cefoperazone sodium-sulbactam sodium + amikacin + metronidazole for 4 weeks, followed by 2 weeks of ciprofloxacin oral treatment). For CVST, we changed to oral anticoagulant therapy (rivaroxaban 10 mg/day). After 1 month of treatment, the follow-up was performed again. Re-examination of enhanced hepatic MRI indicated that the scope of the right lobe abscess was reduced compared with that of the previous one (Fig. 4A-D). However, head MRV showed that the right transverse sinus and sigmoid sinus had not changed significantly (Fig. 5 A-D).

## Discussion and conclusion

In this study, we reported a rare case of *Klebsiella pneumoniae* primary liver abscess with CVST. The patient developed transient brain dysfunction after chills and fever and was diagnosed as liver abscess by abdominal CT and enhanced liver MRI. CVST was confirmed by the combination of enhanced cranial MRI and cranial MRV. The patient recovered well after receiving antibiotics and antithrombotic drugs.

Thrombophlebitis of the portal or hepatic venous systems could be a predisposing condition to metastatic disease for liver abscess [13]. In a retrospective study of 169 patients with KLA in Singapore, thrombophlebitis was identified in 53/169 (31.4%); of those, 3 (1.77%) were affecting portal vein, 49 (28.9%) hepatic vein and 1 (0.59%) inferior vena cava (IVC) [14]. While, CVST in patient with KLA has been rarely reported. CVST is a rare but severe complication of bacterial meningitis. In a prospective nationwide cohort study of bacterial meningitis in the Netherlands, 26 (1%) of 2220 adults with community-acquired bacterial meningitis complicated with CVST [15]. The symptoms and signs of CVST and bacterial meningitis are similar, so that the identification of bacterial meningitis complicating CVST is a challenging task [10, 16, 17]. In our case, we cannot completely rule out the presence of bacterial meningitis because the patient was treated with antibiotics prior to LP. According to the guidelines [18, 19], patients with suspected bacterial meningitis who have none of the following risk factors (e.g., immunocompromise, history of central nervous system disease, papilledema, focal neurologic deficit, new onset seizure, altered consciousness) should be emergently performed blood cultures and LP prior to antibiotic treatment. This reminds us that when patients with superior brain function disturbances, immediate and appropriate examination is needed to avoid misdiagnosis of meningitis complicated with a CVST.

There are also some reports of CVST with other site infections, such as abdominal wall abscess [20], appendiceal abscess [21] and miliary tuberculosis [22]. The risk factor for CVST in our case was liver abscess. Systemic inflammatory response caused by liver abscess produced a large number of inflammatory factors, leading to coagulation cascade activation, platelet activation and plasminogen activation. Vascular endothelial injury caused by inflammation was also involved, which jointly caused hypercoagulability and made patients susceptible to CVST. Therefore, we speculate that liver abscess may be the cause of thrombosis in this patient, which leads to CVST. However, the relationship between liver abscess and CVST and its pathophysiological mechanism still need further research.

CVST has a highly variable clinical manifestations and the median time from the onset of clinical symptoms to the final diagnosis was 7 days [10]. International Study on Cerebral Vein and Dural Sinus Thrombosis (ISCVT) reported, including 624 patients, the following as the most common symptoms: headache (88.8%), epilepsy (39.3%), paresis (37.2%), optic papilledema (28.3%), and altered mental status (22%) [10]. The case we reported did not present with the usual headache, but with transient altered mental status. No matter what, patients with KLA present clinically unexplained headache, epilepsy, altered mental status or other neurological disorders, clinicians need to consider the possibility of CVST, especially for those who have high risk factors for CVST or with hypercoagulable condition.

It is difficult to establish the diagnosis of CVST in majority of cases, because of its extensiveness and high variability of clinical manifestations. Therefore, neuroimaging examination is crucial. The initial imaging technique to assess patients with CVST is usually a CT scan of the brain. However, due to the low sensitivity of CT, only 30% of CVST patients are positive [23], and more than 20% of CVST patients were completely normal [24]. Therefore, for patients with suspected CVST, the most sensitive neuroimaging currently recommended is a combination of cerebral contrast-enhanced MRI and CTV/MRV [25–27]. In this case, the combination of MRI and MRV results helped us complete the final diagnosis of CVST.

According to the recommendations of recent guidelines [25, 26], once CVST is diagnosed, treatment should be immediate, including reversal of the known underlying etiology, early and standardized anticoagulation therapy, intravascular therapy, other drug therapy and treatment of complications. LMWH is the preferred drug in the acute phase, and oral anticoagulants should be continued after the acute phase, with a recommended duration of 3–12 months to prevent recurrence and other venous thromboembolic events [26]. In this case, the anticoagulant therapy of LMWH was given to the patient at the first time after the CVST diagnosis, and at the same time, sufficient courses of antibiotics were given to reverse the cause of infectious CVST. After the above comprehensive treatment, the patient's infection was controlled and there was no recurrence of headache, seizures or mental status changes. Follow-up one month later, the patient's liver abscess was significantly smaller than before, but the right transverse sinus and sigmoid sinus thrombosis did not change much. This may be because the full course of oral anticoagulant therapy has not reached and may require longer follow-up to determine the patient's outcome.

KLA is the most common but dangerous type of liver abscess, which can often lead to “invasive syndrome” and many complications, but complicated with CVST is extremely rare in clinic. The purpose of this case report is to alert physicians to pay attention to this rare complication and to emphasize appropriate investigation as soon as possible to rule out bacterial meningitis. Multidisciplinary cooperation in diagnosis and treatment will benefit such patients. However, the relationship between liver abscess and CVST and its pathophysiological mechanism still need further research.

## Data Availability

All data generated and analyzed in this study are included in this article.

## Abbreviations

CVST: Cerebral venous sinus thrombosis
KLA: *Klebsiella pneumoniae* Primary liver abscess
CT: Computed tomography
MRI: Magnetic resonance imaging
MRV: Magnetic resonance venography
MODS: Multiple organ dysfunction syndrome
ISCVT: International studies on Cerebral Vein and Dural Sinus Thrombosis
WBC: White blood cell count
NEUT%: Neutrophil ratio
PLT: Platelets
CRP: C-reactive protein
DDI: D dimer
SCr: Serum creatinine
PCT: Procalcitonin
ALT: Alanine aminotransferase
AST: Aspartate transaminase
TBIL: Total bilirubin
LMWH: Low-molecular-weight heparin
BUN: Blood urea nitrogen
ALB: Serum albumin
INR: International normalized ratio
TnT: Troponin T
NT-pro BNP: N-terminal pro-brain natriuretic peptide
LAC: Lactic acid
CSF: Cerebrospinal fluid
LP: Lumbar puncture
